# The “Faculties” and Honorary Degrees

**Published:** 1895-10

**Authors:** 


### THE "FACULTIES" AND HONORARY DEGREES.

The history of honorary degrees has never yet been written, but if ever prepared it should show one of the most interesting phases of poor, weak human nature. The average credulous mind invariably takes it for granted that when the announcement is made that Mr. —— has had the degree of M.D., D.D.S., A.M., or LL.D. conferred without curriculum that it is an evidence that Faculties have been impressed with the great skill or learning of the individual and have conferred the honor in kindly recognition of the fact. That this is rarely true is well understood. Influences are brought to bear to an extent not generally appreciated. So much is this the case that those familiar with the subject have long since ceased to feel otherwise than a measure of contempt for such honors no matter from whence derived.

The Association of Faculties early set its face sternly against any such degree, and supposed it had thoroughly barred the way beyond which there could be no trespassing. At the recent meeting at Asbury Park the effort was made to have it conferred, and it came very near being successful. We are disposed to believe that the majority vote permitting such action was the result of thoughtlessness in some of the delegates present, which upon subsequent consideration led to a reconsideration of the vote and rejection of the application.

The possibility of this attempt being eventually successful should lead this Association to pass a law, at its next session, making it absolutely impossible for any of the separate colleges, departments of universities, or the universities themselves to grant the degree of Doctor of Dental Surgery or its equivalent. The attempt to influence this Association and other recent occurrences should be warnings to be heeded. The dental profession of America cannot afford to peril its reputation by countenancing in any way this degree.

The course of some of the members of the Association of Faculties in this matter is not comprehensible in the light of their public utterances, but this is only another evidence that some men seem to prefer retrograde movements rather than a perpetual advance towards a more perfect standard.

It is very satisfactory to note that the American Dental Association placed itself positively on record upon this subject, and we believe this expression will prevent all future efforts in this direction.

